# Large Language Models in Clinical Neurology: A Systematic Review

**DOI:** 10.21203/rs.3.rs-8902070/v1

**Published:** 2026-02-18

**Authors:** Alon Gorenshtein, Kamel Shihada, Mahmud Omar, Yiftach Barash, Girish N Nadkarni, Eyal Klang

**Affiliations:** 1. Department of Neurology, Harvard Medical School, Boston, MA; 2. Department of Neurology, Beth Israel Deaconess Medical Center, Boston, MA; 3. The Windreich Department of Artificial Intelligence and Human Health, Mount Sinai Medical Center, NY, USA.; 4. The Hasso Plattner Institute for Digital Health at Mount Sinai, Mount Sinai Health System, NY, USA.; 5. Azrieli Faculty of Medicine, Bar-Ilan University, Safed, Israel.; 6. Division of Vascular and Interventional Radiology, Department of Radiology, Beth Israel Deaconess Medical Center, Harvard Medical School, Boston, MA, USA; 7. The Charles Bronfman Institute for Personalized Medicine at Mount Sinai, Icahn School of Medicine at Mount Sinai, New York City, New York, USA

**Keywords:** Neurology, AI agent, Large language model, Systematic Review, AI

## Abstract

**Background:**

Large language models (LLMs) are increasingly explored for clinical applications in neurology, yet their real-world utility, safety, and optimal implementation remain uncertain. We systematically reviewed the literature to characterize current applications, evaluate evidence quality, and identify knowledge gaps regarding LLM use in clinical neurology.

**Methods:**

Following PRISMA guidelines, we searched PubMed, Embase, Scopus, Web of Science, and CENTRAL from January 1, 2022 through February 1 2026. for peer-reviewed studies evaluating LLM applications in clinical neurology. We included studies using large language models for clinically relevant neurology tasks from text or multimodal inputs. Two independent reviewers screened records, extracted data, and assessed risk of bias using the QUADS-AI. We synthesized evidence narratively across application domains, validation approaches, and model performance.

**Results:**

Thirty-six studies (published 2023–2026) spanning 8 neurology subspecialties met inclusion criteria; 13 were simulation or feasibility studies, 17 analyzed retrospective clinical data, and 6 reported prospective clinical validation. Proprietary models predominated; 7 studies used retrieval-augmented generation (RAG) and 3 used agentic frameworks. Performance was highest for constrained tasks, including binary diagnostic classification (area under the curve, AUC 0.75–0.94), information extraction (F1 score, 0.89–0.90), patient education question answering (accuracy, 68%−97%), and ischemic stroke thrombectomy decision support (AUC, 0.92). Open-ended case-based classification showed lower accuracy (42%−54%). Safety signals included hallucinations and fabricated citations, overconfident recommendations, and poor calibration; risk of bias was rated high in all included studies.

**Conclusion:**

LLMs show promise for selected neurology workflows, but current evidence is early, heterogeneous, and limited by high risk of bias and scarce prospective validation. Clinical translation will likely require RAG and agentic architectures that can plan multi-step tasks, retrieve guidelines and local protocols, verify and calibrate outputs, and produce structured, auditable recommendations with source attribution, with clinician oversight and prospective evaluation.

**Primary Funding Source:**

This work was supported in part through the computational and data resources and staff expertise provided by Scientific Computing and Data at the Icahn School of Medicine at Mount Sinai and supported by the Clinical and Translational Science Awards (CTSA) grant UL1TR004419 from the National Center for Advancing Translational Sciences. Research reported in this publication was also supported by the Office of Research Infrastructure of the National Institutes of Health under award number S10OD026880 and S10OD030463.

**Registration:**

PROSPERO CRD420251082465

## INTRODUCTION

The growing integration of artificial intelligence (AI) into medical practice is transforming healthcare delivery.^1^ Large language models (LLMs), such as Chat Generative Pre-trained Transformer (ChatGPT), can interpret and generate complex clinical text and are being explored for tasks including clinical decision support, documentation assistance.^2^

Neurology is a particularly demanding field for these tools.^3^ Neurological disorders are highly prevalent and contribute substantially to the global burden of disease, generating heavy workloads for physicians and healthcare systems.^4^ At the same time, neurological diagnosis often hinges on detailed linguistic descriptions of symptoms and examination findings that must be interpreted alongside multimodal data, e.g. neuroimaging and electrophysiology.^5^

Early studies nevertheless suggest that LLMs may add value in selected neurology-related applications.^6^ Be it as it may differences in task framing, prompting strategies, data sources (synthetic versus real-world clinical data), evaluation metrics, and clinical comparators make results difficult to compare across studies and limit generalizability.^7^ This variability is not only a methodological challenge but also a safety concern. In high stake field as neurology, these models pose risk as they may generate confident but incorrect or fabricated statements (“hallucinations”), propose misleading differential diagnoses, or suggest inappropriate management steps, including medication choices.^8^ Risks may be amplified when models are used outside well-defined use cases or without human oversight, and performance can further degrade in rare syndromes, atypical presentations, or multilingual documentation.^9,10^

Taken together, these factors highlight the need for a systematic review that synthesizes the current literature into a cohesive evidence map for neurologists. Such a review can clarify where LLMs perform reliably, where evidence is limited or inconsistent, how studies are being evaluated, and what safety risks and implementation gaps remain before broader clinical adoption.

## METHODS

### Protocol and reporting

We conducted this systematic review in accordance with PRISMA 2020. A protocol was prepared a priori and registered in PROSPERO (CRD420251082465).

### Conceptual framework and operational definition

We evaluated large language model (LLM) applications in clinical neurology. An LLM application was defined as a generative transformer-based model used during inference to perform a clinically relevant neurology task from text and or multimodal inputs (for example clinical histories and physical examinations, imaging reports, free-text clinic letters, patient narratives, transcripts, paired imaging, or structured questions). We included both proprietary and open-weight models, and both standalone LLM pipelines and hybrid systems, including retrieval-augmented generation (RAG), multi-agent orchestration, and LLM plus conventional machine learning ensembles, provided the LLM materially contributed to the evaluated output. Systems focused solely on non-generative NLP without an LLM at inference, lack of clinical relevancy, benchmark studies without integration of models beyond Base LLM were excluded.

### Data sources and search strategy

We searched PubMed, Scopus, Web of Science, and CENTRAL for peer-reviewed studies from January 1st, 2022 through Febubary 1st 2026. Searches were restricted to English-language records. The PubMed strategy used concept blocks capturing (1) LLMs and generative AI and (2) clinical neurology domains and tasks. Search terms included large language model, LLM, generative AI, ChatGPT, GPT, Claude, Gemini, Bard, Llama, Mistral, and neurology terms spanning stroke and vascular neurology, epilepsy, neuroimmunology, movement disorders, neuro-oncology, neuromuscular medicine, headache, and language disorders. The strategy was translated to other databases using database-specific field tags while preserving the concept structure. Reference lists of included studies and relevant reviews were screened to identify additional eligible articles. Full search startgery in supplementary appendix.

### Eligibility criteria

We included peer-reviewed original studies that evaluated an LLM-based system on a clinical neurology task, including diagnostic classification, differential diagnosis generation, neuroanatomical localization or phenotyping, clinical decision support and guideline adherence, prognosis or monitoring, clinical documentation and summarization, and information extraction from clinical text. Studies were required to report an empirical evaluation using quantitative metrics, structured human evaluation, or both.

We excluded non-original publications (reviews, editorials, protocols), conference abstracts without a peer-reviewed full text, studies outside clinical neurology, studies that did not use an LLM during inference, and studies without evaluative outcomes.

### Study selection

After deduplication, two reviewers independently screened titles and abstracts and then reviewed full texts for eligibility. Discrepancies were resolved by discussion and consensus.

### Data extraction and data items

We used a standardized extraction template. Study-level variables included year, country, clinical domain, setting, study design, sample size, data source type (real-world clinical data, synthetic data, vignettes, benchmark questions), input modality (text-only vs multimodal), primary task, model name and version, and whether the system used augmentation (RAG, tools, agents, or hybrid ML).

Outcomes extracted included primary performance metrics (for example AUC, sensitivity and specificity, accuracy, F1, agreement statistics, or rubric-based grades), comparator performance when available (clinicians, conventional ML or NLP, rules, or LLM-only baselines), and implementation characteristics when reported (prompting approach, latency, cost, reproducibility). We also extracted explicitly reported safety considerations and failure modes, including hallucination risk, citation fabrication, over-calling or low specificity, semantic false positives, numerical reasoning failures, and performance gaps in rare clinical subgroups or anatomical regions.

### Task taxonomy and classification

To support synthesis, we categorized each study into prespecified task categories aligned with clinical use: diagnostic classification, information extraction, patient education and communication, clinical decision support, localization or phenotyping, and prognosis or monitoring. When a study evaluated multiple tasks, it was assigned to each relevant category for qualitative synthesis, while preserving the primary task as reported by the authors.

### Outcomes and synthesis

The primary outcome was task performance relative to the study-defined reference standard and comparator, where applicable. Secondary outcomes included safety signals and failure modes, auditability and traceability features (for example citations, guideline tethering, or structured outputs), and operational feasibility. Due to heterogeneity in tasks, datasets, metrics, and evaluation designs, we did not perform a meta-analysis. We conducted a structured narrative synthesis with descriptive statistics, summarizing performance ranges within task categories and highlighting consistent patterns of success and limitation across domains and study designs.

### Risk of bias and applicability assessment

We adapted the QUADAS-AI tool^11^ (Quality Assessment of Diagnostic Accuracy Studies for AI) to assess risk of bias and applicability concerns. This modified instrument evaluated four domains: (1) Patient/Data Selection, (2) Index Test (AI system), (3) Reference Standard, and (4) Flow and Timing. Each domain was rated as low risk, high risk, or unclear risk of bias.34 Additionally, we assessed concerns regarding applicability for the first three domains. Specific high-risk indicators included: use of synthetic/simulated data only, single-center validation, sample size <100, lack of external validation, unclear ground truth establishment, and selective outcome reporting. Two reviewers independently assessed each study, with disagreements resolved through consensus discussion.

## Results

### Study Selection and Characteristics

**Figure 1.**
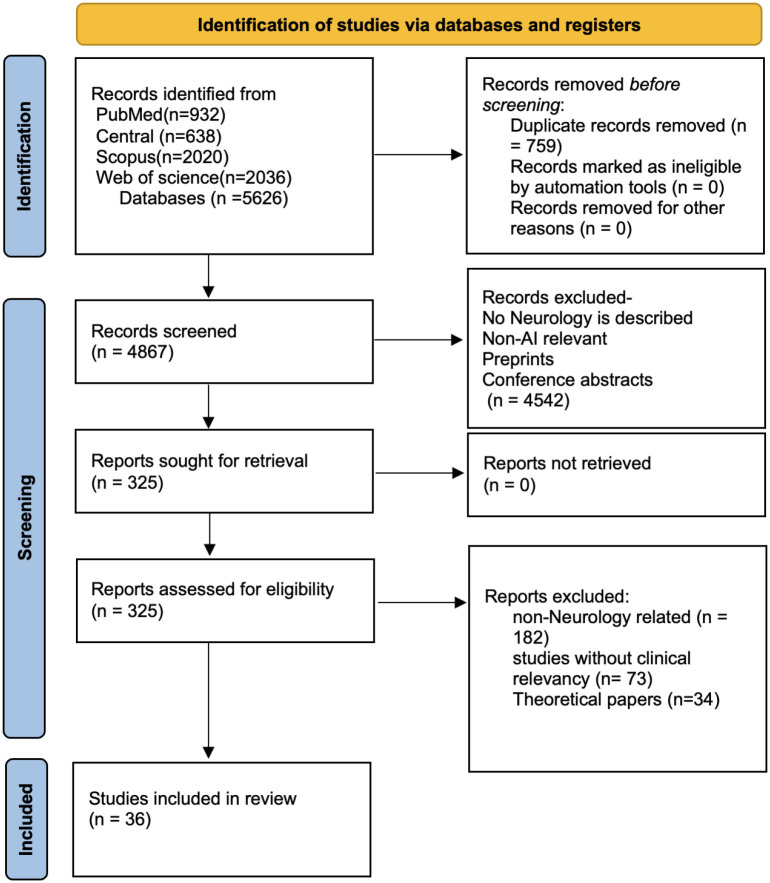
Study identification and selection PRISMA 2020 flow diagram of study selection for the systematic review of neuro-symbolic large language model (LLM) systems in clinical medicine. Database searches identified 3,166 records (PubMed/MEDLINE n=1,021; CENTRAL n=643; Scopus n=38; Web of Science n=1,464). After removing 232 duplicates, 2,934 records were screened and 121 full-text reports were assessed for eligibility. Twenty-one studies were included. Full-text exclusions were due to non-neuro-symbolic LLM systems (n=82), lack of clinical relevance (n=14), or theoretical papers without downstream clinical evaluation (n=4).

We identified 5,626 records. After removing 759 duplicates, 4867 records were screened; 325 full texts were assessed; and 36 studies met inclusion criteria (Figure 1).^12–47^ Studies were published between 2023 and 2026, with 1/36 (2.8%) in 2023, 9/36 (25.0%) in 2024, 25/36 (69.4%) in 2025, and 1/36 (2.8%) in 2026, reflecting the rapid emergence of this field. Clinical domains spanned 8 neurological subspecialties: epilepsy (n=9, 25.0%), general neurology (n=9, 25.0%), stroke/vascular neurology (n=8, 22.2%), neuro-immunology (n=3, 8.3%), movement disorders (n=2, 5.6%), neuromuscular disease (n=2, 5.6%), neuro-oncology (n=2, 5.6%), and neurocritical care (n=1, 2.8%). Study designs varied in rigor: 13/36 (36.1%) were simulation/feasibility studies, 17/36 (47.2%) analyzed retrospective clinical data, and only 6/36 (16.7%) achieved prospective clinical validation, representing a critical translational gap (Table 1). GPT-4 variants (GPT-4, GPT-4o, GPT-4-Turbo, GPT-4-Vision) were the most frequently employed LLMs, appearing in at least 6/36 studies (16.7%) as primary models, with additional studies using GPT-4 alongside other models. Proprietary/closed-source models dominated (25/36, 69.4%). Retrieval-augmented generation (RAG) was employed in 7/36 studies (19.4%), multi-agent frameworks in 3/36 (8.3%), while open-source models, including Llama, Mistral, Gemma-2, and BERT variants were evaluated in 9/36 studies (25.0%). Retrieval-augmented generation (RAG) was employed in 7/36 studies (19.4%), and multi-agent frameworks in 3/36 (8.3%).

### Risk of bias

Overall risk of bias was rated high in all 36 studies. This was driven extensively by participant selection, which was rated high risk in 36/36 (100%) studies. This high risk stems from the fact that the majority of evaluations relied on single-center cohorts without external validation, small sample sizes (<100), or synthetic/simulated data (e.g., vignettes, exam questions, or public datasets) rather than real-world clinical streams. These selection biases also generated high applicability concerns across the cohort. Analysis limitations remained a major driver of bias, with most studies failing to report calibration or clinical-utility metrics, relying instead on discrimination metrics (e.g., Accuracy, AUC) derived from internal validation alone. In contrast, the index test and reference standard domains were generally rated lower risk, although specific concerns regarding data leakage (e.g., models trained on public cases used for testing) and unclear ground truth were identified in a subset of studies (Supplementary Methods Section 4).

### Clinical Domain Distribution

Task applications varied by domain. Epilepsy studies focused predominantly on diagnostic classification (3/9) and patient education (2/9), with additional work in information extraction for anti-seizure medication documentation and epileptogenic zone localization. General Neurology encompassed the broadest task diversity, including diagnostic reasoning, summarization, and clinical decision support. Stroke/Vascular Neurology studies addressed the full clinical pathway from screening and localization to prognostic prediction. The two Movement Disorders studies both leveraged LLM-derived features from speech transcripts for Parkinson’s disease staging, while Neuromuscular studies focused on electrodiagnostic report interpretation and generation. Validation rigor also varied by domain (Supplementary Figure X). Notably, Stroke/Vascular Neurology despite being among the most studied domains had no prospective clinical validation studies (0/8), with 5/8 retrospective and 3/8 simulation designs. In contrast, smaller domains achieved higher validation rates: Movement Disorders (1/2 prospective), Neuromuscular (1/2 prospective), and Neuro-oncology (1/2 prospective). The six prospective studies spanned epilepsy diagnosis (Brigo et al.), neurocritical care guideline adherence (Kliem et al.), patient education benchmarking (Li et al.), Parkinson’s motor state classification (Castelli), neuro-oncology decision support (Tini et al.), and EMG/NCS report generation (Gorenshtein 2025_a et al.).

### Architectural Determinants of Performance

**Figure 2.**
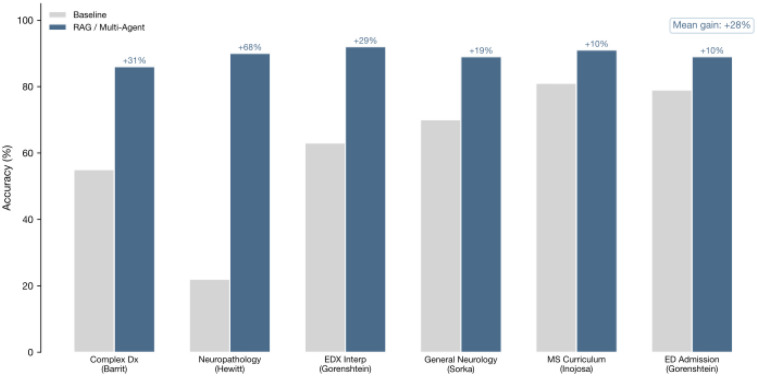
Performance Comparison of Large Language Model Architectures in Clinical Neurology Applications Legend: A comprehensive explanation of the figure showing RAG-enhanced systems, multi-agent frameworks, and open-source models, with specific performance improvements across neuropathology classification, differential diagnosis, and other tasks. Includes details about the +28 percentage point mean improvement.

Model architecture substantially influenced outcomes (Figure 2, Table 3). RAG was employed in 7/36 studies (19.4%) and consistently outperformed standard prompting: neuropathology classification improved from 22% to 90% with RAG (Hewitt et al. 2024);^39^ complex differential diagnosis rose from 55% (neurologists) to 86% (RAG-GPT4) (Barrit et al.);^45^ MS curriculum performance increased from 81% to 91% (Inojosa et al.).^32^ Multi-agent frameworks (3/36, 8.3%) showed comparable gains: INSPIRE 3-agent improved EDX interpretation from 62.6% to 92.2% (Gorenshtein et al. 2025_d);^13^ CrewAI boosted LLaMA-3.3–70B from 69.5% to 89.2% on board exams (Sorka et al.).^12^ Mean improvement across enhanced architectures was +28 percentage points (Figure 4). Open-source models (9/36, 25.0%) approached proprietary performance for well-defined tasks: Llama-2–13B achieved F1 ~0.90 for ASM extraction (Fang et al.)^35^; Gemma-2/Llama-3.1 reached 98.4% for Parkinson’s classification (Castelli et al.).^29^

## Safety Signals and Failure Modes

Five systematic failure patterns recurred across studies (Table 3). *Epistemic failures* (hallucination) were acknowledged in 7 studies, with citation fabrication documented: Wu (2024) noted “risk of fabricated citations” in patient education, while RAG-augmented systems with source attribution reported no unverifiable claims (Barrit et al.).^45^ *Calibration deficits* manifested as diagnostic over-calling: Brigo et al. reported 100% sensitivity but 26.7% specificity for epilepsy;^17^ Chen et al. noted “semantic false positives” flagging inactive history as active contraindications;^20^ Maiorana et al. found LLMs over-ordered investigations in 17–25% of cases.^40^ *Action bias* toward intervention was quantified: Shmilovitch (2025) found GPT-4 over-recommended tPA in 11/105 cases (10.5%); Tini et al documented radiotherapy mismatches in gray-zone glioma cases despite clinical equipoise.^18^ *Numerical reasoning failures* emerged: Acır et al. showed standalone GPT-4 had r=0.054 for NIHSS prediction, improving only with structured ML integration (r=0.513).^44^ *Domain knowledge gaps*: cerebellar localization was consistently less accurate than supratentorial (Lee et al.);^37^ epileptogenic zone identification failed for cingulate and insular regions (Luo et al.).^28^ Studies positioning LLMs as autonomous decision-makers consistently reported concerning safety signals, while working as a “copilot” showed improved outcomes. AI-alone achieved quality score 0.70 versus 0.94 for AI+physician, identical to physician-alone (Gorenshtein 2025_a^15^, Table 4).

## Discussion

**Figure 3.**
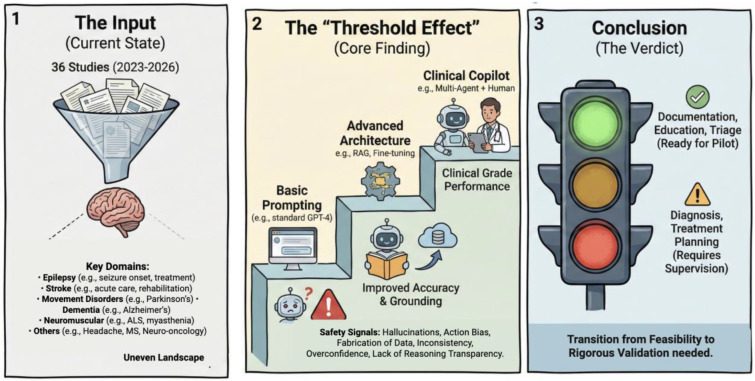
Central Illustration. The Landscape, Threshold Effect, and Clinical Readiness of Large Language Models in Neurology. (A) The Landscape. Distribution of the 36 included studies (2023–2026) across clinical domains, illustrating the dominance of Epilepsy and Stroke research compared to underexplored subspecialties (e.g., Neuromuscular, Neuro-oncology). (B) The Threshold Effect. Comparative performance of LLM architectures. Basic prompting (bottom step) is associated with critical safety signals (hallucination, action bias). Enhanced architectures such as Retrieval-Augmented Generation (RAG) and Multi-Agent systems are required to cross the performance threshold, with the “Clinical Copilot” (Human-AI collaboration) model representing the current peak of safety and efficacy. (C) Clinical Readiness. Traffic-light classification of current viability based on safety profiles: Documentation and Education are ready for pilot implementation (Green); Triage and Screening require strict human oversight (Yellow); and Autonomous Diagnosis remains unsafe due to reliability concerns (Red).

Our findings indicate that LLM architectures demonstrate promise in neurology; Be it as it may, the methodologies employed in most studies and identified safety concerns suggest these models are not yet suitable for clinical deployment. A total of 36 studies were identified, spanning eight neurological subspecialties. Performance was encouraging in diagnostic classification (AUC 0.75–0.92), information extraction (F1 0.85–0.90), and patient education (accuracy 68–97%). However, only 16.7% of studies achieved prospective validation, and just 5.6% demonstrated external generalizability. This disparity between technical potential and clinical readiness constitutes the pressing challenge in the field (Figure 3.).

The safety signals identified in this review are concerning espeically at high stake domain such as neurology. LLMs are trained to generate plausible, helpful responses, an optimization target that creates systematic bias toward action over appropriate restraint. In stroke care, this manifested as thrombolysis over-recommendation in cases where experts recognized contraindications; in neuro-oncology, radiotherapy was endorsed in gray-zone glioma scenarios where watchful waiting may be equally valid. These are not random errors but predictable consequences of training objectives that reward confident, interventional outputs.^48^ The clinical stakes are profound: hemorrhagic transformation from inappropriate thrombolysis and radiation necrosis from unnecessary treatment represent precisely the irreversible harms that neurological training teaches clinicians to prevent through careful equipoise. The calibration deficits we observed, 100% sensitivity paired with 27% specificity for epilepsy diagnosis reflect the same underlying asymmetry.^49^ LLMs appear optimized for recall at the expense of precision, generating false positives^50^ that translate directly into unnecessary antiepileptic drug exposure, patient anxiety from unwarranted diagnostic labels, and healthcare resource utilization from excessive testing. Perhaps most concerning, domain knowledge gaps followed an inverse relationship with clinical need: cerebellar localization was consistently less accurate than supratentorial lesions, and epileptogenic zone identification failed specifically for cingulate and insular regions. This pattern likely reflects training data imbalances where common presentations dominate the corpus while rare but clinically important entities remain underrepresented^51^ meaning current LLMs may reinforce rather than address the diagnostic challenges most difficult for neurologists. These vulnerabilities extend beyond our neurology-specific findings. Omar and colleagues demonstrated that LLMs are “highly vulnerable to adversarial hallucination attacks,” with 50–82% hallucination rates when fabricated clinical details were embedded in clinical vignettes and prompt-based mitigation failed to eliminate this risk.^52^ The implication is sobering: base LLMs lack the deep relational understanding necessary for the anatomical, syndromic, and prognostic reasoning that characterizes neurological expertise. This limitation is architectural, not addressable through prompt engineering or temperature adjustments, and demands fundamentally different approaches to clinical deployment.

A path forward for mitigating these safety issues lies in retrieval-augmented generation (RAG) and agentic systems. RAG is an architectural approach that augments LLM generation with retrieved documents from external knowledge bases, grounding model outputs in verifiable sources rather than relying solely on parametric knowledge embedded during training.^53^ This enables real-time access to current guidelines, institutional protocols, and domain-specific literature while providing source attribution that facilitates verification. AI agent systems extend this paradigm further, these are architectures that can plan multi-step tasks, invoke external tools (including RAG retrieval, calculators, and database queries), and in some cases coordinate reasoning across multiple specialized agents.^54^ The evidence from our included studies supports these architectural approaches. RAG-augmented systems reported no unverifiable claims while substantially improving diagnostic performance, neuropathology classification accuracy rose from 22% to 90% when LLMs were grounded in retrieved pathology literature. Multi-agent frameworks demonstrated comparable gains through orchestrated reasoning: the INSPIRE three-agent system (Validator, Knowledge Integrator, Synthesizer) improved electrodiagnostic interpretation from 62.6% to 92.2%, while multi-agent decomposition transformed LLaMA-3.3 from 69.5% to 89.2% on neurology board examinations, elevating a mid-tier model to near-expert performance. When baseline model knowledge is insufficient, agentic workflows use targeted retrieval of primary medical sources to close information gaps and ground outputs in evidence rather than parametric recall.^55^ For quantitative prediction, hybrid architectures that combine LLM reasoning with structured machine learning models improved NIHSS prognostication from r=0.054 (functionally no association) to r=0.513 (moderate predictive value). That all six prospective validation studies in our corpus employed these enhanced architectures rather than basic prompting suggests that architectural augmentation may be prerequisite rather than optional for neurological applications meeting clinical-grade standards (Figure 3.).

However, the agentic systems proposed to mitigate base LLM failures may introduce novel failure modes requiring independent evaluation. As AI agents gain tools they gain the capability of autonomy, such autonomy come with it’s own price. Klang et al. tested clinical agents in EHR workflows and found they demonstrated “indifference” to patient identity inconsistencies accurately coding encounters but writing them into incorrect patient records.^56^ Agents failed to detect subtle tampering that human verification would flag: single-digit MRN changes passed unnoticed in nearly all cases. In neurology, such misbinding failures could result in stroke protocols applied to wrong patients, seizure medications reconciled to incorrect records, or genetic testing results misattributed across families, errors with cascading consequences for treatment decisions and inherited disease counseling. Similarly Agentic LLMs treated patient tone as clinical input, altering triage, follow-up, prescribing, and sick-leave decisions despite identical symptoms.^57^ These findings suggest the field must evaluate autonomy, abstention appropriateness, alongside accuracy. Safe deployment of neurological AI agents will require explicit verification gates before treatment recommendations, deterministic upstream checks on patient identifiers, and continuous monitoring frameworks.

This review is limited by English-language restriction, heterogeneity precluding meta-analysis, and likely publication bias. QUADAS-AI assessment revealed pervasive methodological concerns: 94% of studies lacked external validation. Rapid model evolution means specific benchmarks require updating, though architectural and safety insights should remain durable.

LLMs represent transformative potential for clinical neurology, yet the gap between technical promise and clinical validation remains striking. The finding that all prospective validation studies employed enhanced architectures whcih none relied on basic prompting suggests a threshold effect: standard LLM deployment may be fundamentally insufficient for neurological applications regardless of model sophistication. Future implementation should prioritize RAG grounding in specialty guidelines, agentic verification systems, and hybrid architectures for quantitative prediction, while recognizing that this emerging infrastructure itself requires safety validation in neurology.

## Figures and Tables

**Table 1.** Study Characteristics, Clinical Scope, and Key Findings

Study	Year	Clinical Domain	Study Design	Sample Size	Primary Task	LLM Model	Main results
**Lee JH**	2024	Stroke/Vascular	Simulation	46	Localization classification	GPT-4	GPT-4 localized stroke from raw H&P with strong side and major-region performance, but weaker cerebellar localization.
**Kottlors J**	2025	Stroke/Vascular	Retrospective	100	Decision support (thrombectomy)	GPT-3	Predicted MT eligibility from CT report + clinical text with **88% accuracy** (AUROC **0.92**), **80% sensitivity** (40/50) and **96% specificity** (48/50); **2/50 FP** and **10/50 FN**.
**Shmilovitch AH**	2025	Stroke/Vascular	Retrospective	100	Decision support + prognosis	GPT-4-1106-preview	Strong agreement with expert AIS treatment decisions (strongest for EVT) and prognostic discrimination for 90-day mortality from text inputs; main discordance was **tPA over-recommendations (n=11)**.
**Acır İ**	2025	Stroke/Vascular	Retrospective	230	Prognostic regression	ChatGPT-4.0 + ML ensemble	Standalone GPT-4 showed **no association** with day-7 NIHSS (r=0.054; p=0.500); GPT-4 + supervised regression correlated with outcomes (r=0.513; p<0.001) and explained **R**^**2**^**=0.263**.
**Chen BY**	2025	Stroke/Vascular	Simulation	150	Information extraction	NeuroGlimpse (LLaMA)	In synthetic notes, an LLM contraindication extractor achieved **F1 ≈ 0.89** and **accuracy ≈ 99%** for thrombolysis contraindications.
**Wang X**	2024	Stroke/Vascular	Retrospective	400	Diagnostic classification (AIS)	GPT-3.5, GPT-4	GPT-4 outperformed GPT-3.5 for AIS and LVO detection (higher AUC/accuracy; fewer reasoning errors); ICH detection remained sensitivity-limited despite high specificity.
**Neo JRE**	2024	Stroke/Vascular	Prospective	280	Patient education	ChatGPT, Google Bard	Stroke-rehab answers were variable with limited local-context specificity; Bard modestly outscored ChatGPT on relevance and safety; low inter-rater agreement (κ).
**Cong Y**	2024	Stroke/Vascular	Retrospective	441	Diagnostic (aphasia)	GPT-2, DistilGPT-2	LLM-based “surprisal” features improved aphasia detection (**F1 ≈ 0.92**) and, with linguistic features, improved subtyping (**F1 ≈ 0.79**), without a strict model-size scaling effect.
**Ford J**	2025	Epilepsy	Retrospective	41	Diagnostic classification	GPT-4	From patient narratives, GPT-4 achieved **57%** balanced accuracy (zero-shot) and **64%** (one-shot), below neurologists (**71%**) but higher in consensus cases (**81%**).
**Brigo F (a)**	2025	Epilepsy	Prospective	37	Diagnostic classification	GPT-4	High sensitivity for seizure presence and strong syndrome and structural-etiology performance, but low specificity for epilepsy and acute symptomatic vs unprovoked (over-diagnosis); experts performed best overall (especially full ILAE classification).
**Luo Y**	2025	Epilepsy	Cross-sectional	852	Decision support / EZ localization	GPT-4	GPT-4 improved EZ localization vs epileptologists (WSens **0.61–0.63** vs **0.49–0.51**), with minimal bias (NPIR ≈ 0), but weaker performance in rare regions (for example cingulate).
**Fang S**	2025	Epilepsy	Retrospective	280	Information extraction (ASM)	Llama-2–13B, Mistral	In clinic letters, Llama-2–13B extracted key epilepsy fields with **F1 up to ~0.90** (strongest for ASMs), though epileptologists achieved the highest overall performance.
**Brigo F (b)**	2025	Epilepsy	Retrospective	597	Diagnostic classification (ED)	ChatGPT-4.0	Binary epilepsy diagnosis showed negligible agreement with neurologists (**κ = −0.01**) and very low sensitivity (**17.6%**), missing most epilepsy cases.
**Wu YX**	2024	Epilepsy	Simulation	378	Patient education	ChatGPT-3.5	Answers were accurate and comprehensive in **~68%** (plus **~12%** correct-but-incomplete) with **~82%** repeatability; strongest for diagnosis/treatment and weakest for prognosis.
**Kim HW**	2024	Epilepsy	Cross-sectional	57	Patient education	ChatGPT-4, ChatGPT-3.5	ChatGPT-4 responses were **~70%** accurate and sufficient, **~28%** accurate but needing clinician detail, and **~2%** mixed outdated/incorrect (0% fully wrong).
**Fennig U**	2025	Epilepsy	Retrospective	56,970	Public health / patient experience	ChatGPT-4 Turbo	LLM labeling of r/epilepsy posts validated (AUC **0.79** epilepsy; AUC **0.77** recent seizure; **99%** age/sex extraction). Theme-based Cox models linked several themes to higher future depression/suicidality forum posting; surgery and driving themes associated with lower risk.
**Goldenholz DM**	2025	Epilepsy	Simulation	240	Information extraction + synthesis	Llama2-13B, Mistral 7B	Multi-LLM extraction recovered simulated RCT efficacy and symptom frequencies with minimal error (**≤3%**).
**Poole S**	2025	Neuro-immunology	Retrospective	14,888	Information extraction (MS)	ChatGPT-4	msLesionprompt converted narrative MS MRI reports into binary indicators (new T2 lesions and enhancing lesions) to enable scalable monitoring from routine text reports.
**Kelly BS**	2025	Neuro-immunology	Retrospective	496	Imaging change detection	GPT-4-Vision	Zero-shot GPT-4V achieved **85% accuracy** for MRI progression, but remained below specialized U-Net/ViT models (~94%).
**Maida E**	2024	Neuro-immunology	Cross-sectional	1,133	Patient communication	ChatGPT-3.5	Among 1,133 respondents with MS, ChatGPT answers were rated more empathetic than neurologists’ with no satisfaction difference; education level influenced preference.
**Galetta K**	2023	General Neurology	Simulation	29	Diagnostic reasoning	GPT-4	On 29 vignettes, GPT-4 top-3 accuracy was **48%** localization and **52%** diagnosis (top-1 **24%**); ancillary data did not improve performance and dropped on harder cases.
**Maiorana NV**	2025	General Neurology	Simulation	28	Diagnostic + test recommendations	ChatGPT-3.5, Gemini	Neurologists outperformed LLMs (**75%** vs ChatGPT **54%** vs Gemini **46%**); LLMs over-ordered tests (**~17–25%**); better in well-defined infectious/neuromuscular cases and worse in ambiguous cases.
**Barrit S**	2025	General Neurology	Blinded comparison	5	Diagnostic reasoning	GPT-4 Turbo + RAG	In 5 complex cases, a RAG-tethered system outperformed 13 neurologists on blinded scoring, produced source-cited outputs with no hallucinations reported, and was faster (~20–30 s vs ~9 min).
**Albaqshi**	2025	General Neurology	Retrospective	56	Diagnostic accuracy (multimodal)	GPT-4v, GPT-4o, Gemini	Best overall was Claude 3.5: **80.4% (45/56)**; best rephrased accuracy **76.8% (43/56)** for text-only and text+image; image-only localization **63.1% (82/130)**; lower temperature increased agreement (κ).
**Inojosa**	2025	General Neurology	Cross-sectional	74	Education benchmarking (MS)	GPT-4o, MS-RAG	MS curriculum Q&A: MCQ accuracy **81.1%** (GPT-4o), **86.8%** (MS-RAG), **91.3%** (Prof. Valmed); open-ended accuracy **66.7%**, **76.2%**, **85%** respectively (Prof. Valmed 0% inaccurate).
**Li L**	2024	General Neurology	Prospective	30	Patient education Q&A	GPT-3.5, GPT-4, Llama2, Claude2	“Appropriate” migraine answers were highest for GPT-4 (**96.7%**), then GPT-3.5/Llama2 (**90%**), Claude2 (**86.7%**), Bard (**83.3%**).
**Gorenshtein A (b)**	2025	General Neurology	Retrospective	1,368	Prediction / decision support	Gemini 1.5 + RAG + ML	Hybrid Neuro-Copilot achieved high admission discrimination (**AUC 0.888**) and aligned with experts despite low expert agreement.
**Gorenshtein A (c)**	2025	General Neurology	Retrospective	250	Summarization / report generation	Gemini 1.5pro + RAG	Generated consult notes preserved meaning (cosine **0.89**) and were more concise; recommendations matched neurologists in **~79%**.
**Sorka M**	2025	General Neurology	Simulation	305	Clinical reasoning	10 LLMs + CrewAI multi-agent	Multi-agent reasoning improved board-style MCQ performance substantially (example: LLaMA-3.3–70B **69.5% → 89.2%**).
**Hewitt KJ**	2024	Neuro-oncology	Simulation	30	Diagnostic classification (neuropath)	ChatGPT-4o, Claude-3.5	**To extract from paper:** evaluates WHO-conformant CNS tumor subtyping from free-text histopathology reports, comparing zero-shot vs RAG linked to WHO guidelines. Your prior “Main results” cell was from a different study.
**Tini P**	2026	Neuro-oncology	Prospective	101	Clinical decision support (RT)	GPT-4	GPT-4 matched most RT decisions but was below human-human agreement and tended to over-recommend RT in gray-zone cases.
**Kliem PSC**	2025	Critical Care	Prospective	NR	Guideline adherence (SE)	GPT-3.5, GPT-4o, GPT-5	More detailed prompts increased guideline concordance; GPT-4o performed best vs GPT-3.5 and GPT-5.
**Crawford JL**	2025	Movement Disorders	Retrospective	100	Diagnostic + severity prediction	Whisper-1	Best PD classification used text-embedding-3-small (accuracy **78% ± 3.5**, AUC **0.78 ± 0.05**); best severity prediction used text-embedding-3-large (RMSE **12.9 ± 1.68**).
**Castelli**	2025	Movement Disorders	Prospective	66	NLP prediction (PD ON/OFF)	Gemma-2, Llama-3.1	Speech ON/OFF classification near-perfect (AUC **0.99**, accuracy **0.984**, 1 error); severity prediction strong (R^2^ **0.68**, ρ **0.81**, RMSE **10.6**).
**Gorenshtein A (a)**	2025	Neuromuscular	RCT	200	Report generation (EMG/NCS)	ChatGPT-4o + RAG	AI-assisted drafting did not outperform usual reporting (AIGERS unchanged); AI-alone was inferior despite good abnormal/normal discrimination.
**Gorenshtein A (d)**	2025	Neuromuscular	Retrospective	219	Report interpretation (EDX)	Gemini 1.5 (3-agent INSPIRE)	Multi-agent INSPIRE outperformed base LLM for EMG/NCS reporting (AIGERS **0.83**; accuracy **92%**).

Abbreviations: AIS, acute ischemic stroke; ASM, anti-seizure medication; ED, emergency department; EDX, electrodiagnostic; EZ, epileptogenic zone; MS, multiple sclerosis; PD, Parkinson’s disease; RAG, retrieval-augmented generation; RT, radiotherapy; SE, status epilepticus; NR, not reported.

**Table 3.** Performance Characteristics of Large Language Models Across Clinical Neurology Task Categories

Task Category	n	Performance Range	Key Success Factors	Key Limitations
Diagnostic Classification	14	Binary: AUC 0.75 to 0.94; Open-ended: 42 to 54%	Structured inputs; RAG improves complex cases (up to +31%)	Low specificity in some settings (for example epilepsy 27%); weaker open-ended differentials
Information Extraction	6	F1 0.89 to 0.90; Sensitivity 90 to 97%	Open-source models can match proprietary; multi-pass pipelines improve consistency	Entity boundary errors on complex regimens and overlapping concepts
Patient Education	7	Accuracy 68 to 97%; empathy often rated higher than physicians	Consistent empathy advantage; scalable Q&A	Citation fabrication; up to 28% require clinician supplementation
Clinical Decision Support	5	Thrombectomy AUROC 0.92; guideline adherence up to 92%	Rule-based decisions translate well; RAG improves guideline concordance	tPA over-recommendation (n=11 in one study); tendency to over-recommend in gray-zone RT cases
Localization/Phenotyping	3	Supratentorial performance up to F1 0.85; EZ WSens 0.61 to 0.73	Clear anatomic descriptions and constrained labels	Lower performance in cerebellar, cingulate, and insular localizations
Prognosis/Monitoring	3	LLM alone r=0.054; LLM+ML r=0.513; PD ON/OFF accuracy 98.4%	Hybrid LLM + ML essential; structured signals improve stability	Standalone prediction can fail; generalization outside training distribution unclear

**Table 4.** Systematic Failure Patterns and Safety Risks of Large Language Models in Clinical Neurology

Failure Category	Definition	Representative Studies	Key Findings	Clinical Implications
Epistemic Failures (Hallucination)	Generation of plausible but fabricated content, including false citations and confident errors on unknown cases	Wu 2024, Li 2024, Galetta 2023, Kim 2024	Citation fabrication in patient education (Wu, Li); confident wrong localization on hard cases with 0% accuracy (Galetta); outdated content in 1.8% of responses (Kim); RAG with source attribution eliminated hallucinations (Barrit, Hewitt)	Misinformation in patient communication; dangerous overconfidence; mitigated by retrieval-augmented generation
Calibration Deficits	Systematic miscalibration between sensitivity and specificity; inability to appropriately express diagnostic uncertainty	Brigo 2025, Chen 2025, Maiorana 2025, Wang 2024, Kottlors 2025	Epilepsy diagnosis: 100% sensitivity, 26.7% specificity (Brigo); semantic false positives flagging inactive conditions as contraindications (Chen); 17–25% investigation over-ordering vs neurologists (Maiorana); 10/50 missed thrombectomy candidates (Kottlors)	Over-diagnosis burden; inappropriate treatment exclusion; unnecessary testing; missed treatment-eligible patients
Action Bias	Systematic preference for therapeutic intervention over conservative management in clinically ambiguous cases	Shmilovitch 2025, Tini 2026, Maiorana 2025, Kliem 2025	tPA over-recommendation in 10.5% of stroke cases (Shmilovitch); radiotherapy over-recommendation in gray-zone gliomas (Tini); excessive workup ordering for functional presentations (Maiorana); variable guideline concordance for status epilepticus (Kliem)	Hemorrhagic transformation risk; radiation necrosis; healthcare cost burden; treatment inconsistency
Numerical Reasoning Failures	Poor performance on quantitative computation including prognostication, dosage interpretation, and severity scoring	Acır 2025, Gorenshtein 2025, Crawford 2025, Wu 2024	Standalone GPT-4 NIHSS prediction r=0.054; improved to r=0.513 with ML integration (Acır); limited nerve conduction value interpretation (Gorenshtein); prognosis avoidance with 55.6% vs 78.6% diagnosis accuracy (Wu)	Unreliable outcome prediction; poor quantitative interpretation; inaccurate prognostic counseling
Domain Knowledge Gaps	Systematic deficits in specific neuroanatomical regions or subspecialties, reflecting training data imbalances	Lee 2024, Luo 2025, Galetta 2023, Maiorana 2025, Hewitt 2024	Cerebellar localization consistently inferior to supratentorial (Lee); cingulate/insular epileptogenic zone failure (Luo); 0% accuracy on hard cases vs 93% on easy (Galetta); zero-shot neuropathology 0% accuracy (Hewitt)	Missed posterior fossa pathology; failure on atypical presentations; inverse correlation between AI performance and clinical need
Autonomous vs Supervised Performance	LLMs add value within cliniciansupervised workflows; autonomous operation degrades quality	Gorenshtein 2025, Sorka 2025, Kelly 2025, Fang 2025	AI-alone quality 0.70 vs AI+physician 0.94 (Gorenshtein RCT); base LLM 62.6% → multi-agent 92.2% accuracy (Gorenshtein INSPIRE); LLaMA 69.5% → 89.2% with CrewAI agents (Sorka); GPT-4V 85% vs purpose-built models 94% (Kelly)	Human oversight required for quality output; agentic architectures required for expert-level performance; simple prompting insufficient for clinical deployment

## Supplementary Material

Supplementary Files

This is a list of supplementary files associated with this preprint. Click to download.

- FullAPPENDIXNeuro.pdf
